# Outcomes of Metal Hypersensitivity in Knee Arthroplasty: A Systematic Review of the Literature

**DOI:** 10.1016/j.artd.2026.102003

**Published:** 2026-04-22

**Authors:** Ryan Roopnarinesingh, Elvire Servien, James Harty

**Affiliations:** a Department of Trauma and Orthopaedic, Cork University Hospital, Cork, Ireland; b Department of Trauma and Orthopaedic, South Infirmary and Victoria Hospital, Cork, Ireland; c Department of Trauma and Orthopaedic, Hospital de la Croix-Rousse, Lyon, Rhône Alpes, France

**Keywords:** Metal implant hypersensitivity, Total knee arthroplasty, Metal allergy, Revision knee arthroplasty, Skin patch testing

## Abstract

**Background:**

Metal implant hypersensitivity remains a subject of ongoing debate in the orthopaedic community. With the global rise in total knee arthroplasty (TKA) procedures per annum and an increasing prevalence of reported metal allergies in the general population, understanding the clinical relevance of metal implant hypersensitivity in knee arthroplasty patients is essential. This systematic review evaluates the relationship between metal hypersensitivity, diagnostic testing, and postoperative outcomes following TKA.

**Methods:**

A literature search was conducted using the Ovid, MEDLINE, and Embase databases for studies published between 1990 and December 2024. Studies assessing diagnosis, management, or outcomes of metal hypersensitivity in TKA were included. Both prospective and retrospective studies were considered. The search yielded 554 records, with an additional 25 studies identified through manual reference screening. Following full-text assessment of 58 articles and application of predefined inclusion and exclusion criteria, 24 studies were included. Data extraction focused on study design, patient demographics, implicated metals, diagnostic methods, interventions, and outcomes. Patient Reporting Items for Systematic Reviews & Meta Analysis (PRISMA) guidelines were followed.

**Results:**

A pooled cohort of 5615 knee arthroplasty cases was analyzed, with a mean patient age of 65.35 years and a female predominance (60.55%). Among these, 1165 patients had a documented history of metal hypersensitivity. Twenty studies reported follow-up durations, with a mean follow-up of 27.59 months. Patient symptoms, diagnostic test results, patient-reported outcome measures, and revision rates were evaluated.

**Conclusions:**

The available literature demonstrates significant heterogeneity and limited comparability across studies. Current evidence suggests that self-reported metal allergy or positive skin patch/lymphocyte transformation tests do not reliably predict poorer pain or functional outcomes following TKA. The clinical significance of metal hypersensitivity remains uncertain, highlighting the need for standardized diagnostic criteria and further high-quality research.

## Introduction

The volume of total knee arthroplasty (TKA) procedures is increasing globally. In the United States, the demand is expected to rise 401% by 2040. Similarly, the Australian Orthopaedic Association National Joint Registry reports an increase of 19.2% in TKA procedures in 2023 [[1], [2], [3]].

Hypersensitivity to a metal implant is controversial and is a diagnosis of exclusion. As with hip arthroplasty, metal ions (cobalt, chromium, and titanium) have been proven to be detected hematogenously following TKA and can exceed normal values even just 1 year postoperative [4,5]. High systemic levels of cobalt and chromium can be concerning for both local and systemic complications, for example, adverse local tissue reactions and cardiac/neurologic toxicity, respectively [6].

Sequelae of implant-associated metal allergy include a spectrum of presentations from cutaneous irritation, pain, poor wound healing, and at worst implant loosening, bony lysis, and failure [[7], [8], [9]]. Some of these same symptoms were highlighted by The National Joint Registry data from the United Kingdom (2024) as some of the top contributing factors for both early and late revisions of TKA [2]. While the role of metal hypersensitivity (MH) and early or late failure in knee arthroplasty remains debatable, it cannot be ruled out entirely, as the symptoms are nonspecific and may mimic other pathological processes.

Dissatisfaction rates for TKA for any cause range between 10 and 20% [10,11]. Interestingly, it has been shown that patient-reported allergies of any kind including MH also have a negative impact on postoperative satisfaction and functional outcome scores [12,13]. Granchi et al. [14] have reported that the rate of MH was higher among patients who had failed total joint arthroplasties in their meta-analysis, however, analyzing other studies would suggest that the consensus is conflicting and the literature, heterogeneous in nature [15].

As such, it remains unclear if there is a true cause-effect relationship between MH and implant failure.

### Prevalence and diagnosis of metal hypersensitivity

The prevalence of metal sensitization on skin patch testing (SPT) in the general population is significant, ranging from 10 to 20%, [16,17] while self-reported MH rates in those with a TKA vary between studies with a range of 4.1%-14% (n = 783) [18,19].

Nickel is the most common sensitivity with a prevalence near 14% followed by cobalt, chromium, titanium, molybdenum, and zirconium [20,21] with women being at a higher risk of sensitization to these metals [7,16].

With this documented prevalence of MH, contrastingly there is a paucity in the diagnostic testing specificity [22,23]. Much of the literature utilizes SPT as it is cost-effective and user friendly [24]. It is recommended as a potential investigation to use in patients with a strong history of MH prior to arthroplasty by the European Society of Contact Dermatitis [25]. However, patch testing has been scrutinized as it triggers a cutaneous reaction, may not mimic a true peri-implant environment [26] and has not been shown to be of practical value in predicting mid-term outcomes of TKA [27]. Lymphocyte transformation testing (LTT) (a blood test that exposes the patient’s lymphocytes and monocytes to metal salts to measure their proliferation using a radioactive nucleotide [3H-thymidine]) for this reason, has been identified as potentially more specific but is more resource-intensive, requires invasive serology, and has a similar false-positive rate as patch testing (PT) [[28], [29], [30]].

To further add to this, there are no unique conclusive histopathological findings from a neo-synovial biopsy post-TKA. Krenn et al. described that the presence of excessive lymphocytic/plasma cellular infiltration, eosinophilic infiltration, or granuloma formation (type 1 membranes) in synovial-like interface membrane tissue samples could suggest implant hypersensitivity, especially with a MH history.

But, with no clear signs/biomarkers on histopathology, tissue diagnosis is of less use in this setting compared with other pathologies [[31], [32], [33]]. HLA genotypes may prove a more sensitive way of assessing the risk for MH development in TKA and could play a role in a revision setting but is yet to be studied on a widespread scale [34,35].

In all, delayed sensitivity is likely a complex interaction of a patient’s gender, genotype, and metal debris volume both locally and systemically.

### Basic science and pathophysiology

Metal implant allergy is typically described as a type IV hypersensitivity reaction. This is a T-cell lymphocyte-mediated immune response which typically takes a few days to a few weeks to occur in a host that has been sensitized to an allergen.

TKA implants, as with all metals, will undergo a certain degree of corrosion when placed in a biological system. Aqueous body fluids are highly corrosive and with concomitant metabolic activity, pitting, crevice, fretting, and galvanic corrosion can occur. Surface fatigue and tribochemical reactions are the main proponents of the prosthetic corrosion. The by-products produced by the corroded metal alloys can inappropriately induce this delayed macrophage-led reaction starting a cascade of events that may culminate in hypersensitivity, chronic inflammation, local tissue destruction, increased bone resorption, and arthrofibrosis [31,[36], [37], [38]].

## Material and methods

### Eligibility criteria

**Figure 1:**
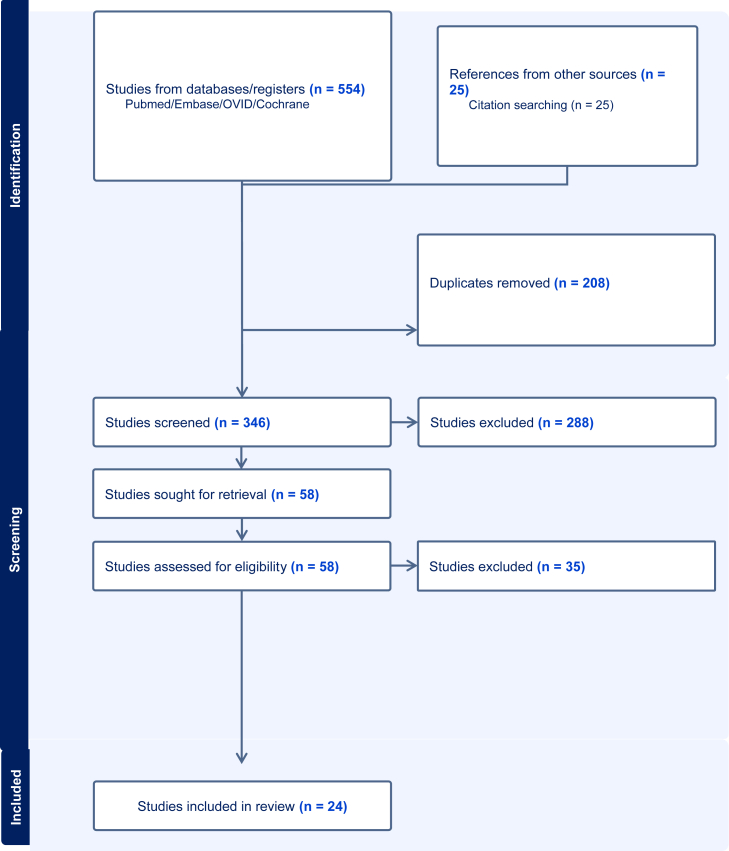
PRISMA flow chart [39].

This review adhered to PRISMA [39] guidelines. PICO strategy was followed: (P) all adult patients undergoing primary or revision TKA; (I) Intervention group comprised of patients with MH or metal allergy; Comparison group comprised of patients without a metal allergy or hypersensitivity; (O) Outcomes included, incidence, prevalence, patient-reported outcome measures (PROMs), and/or the requirement for revision arthroplasty associated with a MH/allergy Figure 1.

### Search strategy and selection criteria

A comprehensive review of the literature (PubMed, Embase, OVID, and Cochrane) was conducted from multiple electronic databases from 1990 to December 2024 for studies evaluating TKA patients with MH/allergy. A manual search of the literature was also conducted for the same time frame for completeness.

Search terms included “Allergy” “Metal Hypersensitivity” “Metal Allergy” “Nickel allergy” “Cobalt allergy” “Titanium allergy” AND “Total Knee Arthroplasty/Total Knee Replacement/Knee Arthroplasty” OR “Knee Prosthesis” AND “English” [Language] (Table 1).

**Table 1:** Search terms and results.

Database	Search term	Years	No. search results
PubMed	(("Metal Hypersensitivity" OR "Nickel allergy" OR "Cobalt allergy" OR "Titanium allergy") AND ("Total Knee Arthroplasty" OR"Knee Replacement" OR "Knee Prosthesis") AND "English"[Language])	1990-2025	301
Embase	(("Metal Hypersensitivity" OR "Nickel allergy" OR "Cobalt allergy"OR "Titanium allergy") AND("Total Knee Arthroplasty" OR "Knee Replacement" OR "Knee Prosthesis") AND [english]/lim)	1990-2025	98
OVID	(Metal Hypersensitivity OR Nickel allergy OR Cobalt allergyOR Titanium allergy) AND (Total Knee ArthroplastyOR Knee Replacement OR Knee Prosthesis)	1990-2025	76

### Inclusion criteria

Studies used in this review included randomized controlled trials, prospective or retrospective cohort studies, and case-controlled studies. Studies that included other *total joint arthroplasty* but had an adequate number of TKA with accessible data were also included. Studies with adequately powered unicondylar knee replacement that fit other inclusion criteria were also included.

### Exclusion criteria

Case reports and cohort studies with a TKA population of less than 15 patients from which to draw data. Studies involving implants other than TKA or unicondylar knee arthroplasty (UKA) or materials not relevant to MH. Studies that were not accessible in the English language or that were not available as a whole text.

### Data extraction and data

Two authors independently reviewed the data extracted from the studies. COVIDENCE software was used to filter the literature. If consensus was not reached on an article, a third author’s opinion was sought. The extracted study characteristics included study, time, follow-up, region, study type, number of patients, female sex, what (if any) specific metal allergy testing was used and age.

### Statistical analysis

Results are expressed descriptively in numbers and percentages. STATA software was used for all data analysis.

## Results

The electronic search yielded 554 results with a further 25 studies being identified through references from prior studies. After removing duplicates using COVIDENCE software, 346 papers remained, and abstracts were screened for relevancy and eligibility. From this, 288 papers were excluded. Fifty-eight full-text papers were then analyzed, and based on previously established inclusion/exclusion criteria, a final 24 studies were deemed appropriate for this analysis.

Significant heterogeneity is seen with all the included studies. Many of the populations, interventions, and clinical outcomes differ across the literature but the conclusions of the papers ultimately comment on the impact of MH on TKA outcomes. Therefore, a robust quantitative analyses could not be performed.

### Quality assessment

Included are 3 randomized controlled trials with a Level of Evidence of II.

Ten retrospective cohort studies and 11 prospective cohort studies all of which are level of evidence of III. All the cohort studies were comparative in nature with an average Methodological Index for Non Randomised Studies (MINOR)S score for comparative non randomized studies [40] of 20.33 out of a possible 24, highlighting good methodological quality in the included studies (Table 2).

**Table 2:** Studies level of evidence and MINORS criteria.

Title	Author	Year	Study type	Level of evidence (LE)	MINORS score
Self-Reported Metal Allergy and Early Outcomes After Total Knee Arthroplasty	Schmidt et al. [42]	2019	Retrospective cohort study	III	21/24
No Increased Risk of Knee Arthroplasty Failure in Patients With Positive Skin Patch Testing for Metal Hypersensitivity: A Matched Cohort Study	Bravo et al. [27]	2016	Matched cohort study	III	19/24
Unicondylar Knee Arthroplasty Using Cobalt-Chromium Implants in Patients With Self-Reported Cutaneous Metal Hypersensitivity	Walker et al. [43]	2019	Prospective cohort study	III	20/24
Hypersensitivity Reactions to Metal Implants: Laboratory Options	Carossino et al. [44]	2016	Prospective cohort study	III	19/24
Sensitivity to Implant Materials in Patients With Total Knee Arthroplasties	Granchi et al. [41]	2008	Retrospective case-control study	III	20/24
Females With Unexplained Joint Pain Following Total Joint Arthroplasty Exhibit a Higher Rate and Severity of Hypersensitivity to Implant Metals Compared With Males Implications of Sex-Based Bioreactivity Differences	Caicedo et al. [45]	2017	Retrospective cohort study	III	20/24
Total Knee Arthroplasty in Patients With Hypersensitivity to Metals	Innocenti et al. [46]	2014	Prospective cohort study	III	20/24
A Comparison of Clinical Outcomes After Total Knee Arthroplasty in Patients Who Have and Do Not Have Self-Reported Nickel Allergy: Matched and Unmatched Cohort Comparisons	Siljander et al. [47]	2024	Retrospective cohort study	III	20/24
Do Patients With Hypoallergenic Total Knee Arthroplasty Implants for Metal Allergy Do Worse? An Analysis of Health Care Utilizations and Patient-Reported Outcome Measures	Tidd et al. [48]	2024	Retrospective cohort study	III	20/24
The association between metal allergy total knee arthroplasty, and revision	Münch et al. [49]	2015	Retrospective registry cohort study	III	19/24
Metallic ion release after knee prosthesis implantation: a prospective study	Lons et al. [4]	2017	Prospective cohort study	III	20/24
Metal hypersensitivity and metal ion levels in patients with coated or uncoated total knee arthroplasty: a randomized controlled study	Lutzner et al. [50]	2013	Randomized controlled trial	II	N/A
Patient-Reported Metal Allergy: A Risk Factor for Poor Outcomes After Total Joint Arthroplasty?	Nam et al. [19]	2016	Retrospective cohort study	III	19/24
Prevalence of metal hypersensitivity in total knee replacement	Desai et al. [51]	2019	Prospective/longitudinal cohort study	III	19/24
Screening for symptomatic metal sensitivity: a prospective study of 92 patients undergoing total knee arthroplasty	Niki et al. [52]	2005	Prospective cohort study	III	18/24
A prospective study concerning the relationship between metal allergy and post-operative pain following total hip and knee arthroplasty	Zeng et al. [53]	2014	Prospective cohort study	III	20/24
Nickel allergy does not correlate with function after total knee arthroplasty	Chimento et al. [15]	2025	Prospective case series	III	20/24
Metal sensitivity in patients with orthopaedic implants: a prospective study	Frigerio et al. [54]	2011	Prospective cohort study	III	19/24
Self-reported metal hypersensitivity in patients undergoing unicondylar knee arthroplasty":	Atilla et al. [59]	2020	Retrospective cohort study	III	19/24
No difference in patient reported outcome and inflammatory response after coated and uncoated total knee arthroplasty a randomized controlled study	Tille et al. [55]	2023	Randomized controlled trial	II	N/A
Improved outcomes in patients with positive metal sensitivity following revision total knee arthroplasty	Zondervan et al. [56]	2019	Retrospective cohort study	III	20/24
Patch Test Results and Outcome in Patients with Complications from Total Knee Arthroplasty: A Consecutive Case Series	Sasseville et al. [57]	2019	Retrospective case series	III	18/24
Lymphocyte Transformation Testing (LTT) in Cases of Pain Following Total Knee Arthroplasty Little Relationship to Histopathologic Findings and Revision Outcomes	Yang et al. [30]	2019	Retrospective cohort study	III	19/24
What role does metal allergy sensitization play in total knee arthroplasty revision?	Lionberger et al. [58]	2018	Retrospective cohort study	III	18/24

### Study characteristics

From the 24 studies [4,15,19,27,30,[41], [42], [43], [44], [45], [46], [47], [48], [49], [50], [51], [52], [53], [54], [55], [56], [57], [58], [59]], there were 5615 knee arthroplasty cases to analyze, with a mean age of 65.35 years (range, 35–92) and a 60.55% female prevalence. Twenty studies [4,15,19,30,[43], [44], [45], [46], [47], [48], [49], [50], [51],[53], [54], [55], [56], [57], [58], [59]] have included a mean follow-up period with an average of 27.59 months (range, 12–79 months) and a mode of 12 months.

### Metal hypersensitivity testing

Three studies focused on patient self-reporting MH with a questionnaire. Both Schmidt and Atilla reported no significant differences in functional outcome in patients reporting self-reported metal allergy (SRMA) on a questionnaire than those who do not. This is contrasted, though, by Nam et al. who related SRMA and lower Knee Society Scores (KSSs) with regard to function and concluded that metal allergy may be associated with poorer functional outcomes after TKA (Table 3) [19,42,59].

**Table 3:** Summary of outcomes of each study.

Title	Author	Outcome
Self-Reported Metal Allergy and Early Outcomes After Total Knee Arthroplasty	Schmidt [42]	The results indicated no significant differences in Knee Society Scores between patients with and without SRMA, suggesting that self-reported metal allergies did not adversely impact early functional outcomes. However, there was a statistically significant difference in WOMAC pain scores, with patients having SRMA reporting a higher mean pain score.
No Increased Risk of Knee Arthroplasty Failure in Patients With Positive Skin Patch Testing for Metal Hypersensitivity: A Matched Cohort Study	Bravo et al. [27]	The findings reveal that patients with SPT+ did not experience significantly higher rates of complications, reoperations, or revisions compared to SPT− patients or matched controls. Specifically, the 5-year survivorship free from revision was notably high across all groups, indicating no substantial difference in outcomes related to metal hypersensitivity. Additionally, postoperative pain levels were comparable between SPT+ and SPT− patients. The study concludes that positive skin patch testing for metal hypersensitivity does not predict adverse outcomes post-TKA, suggesting that preoperative metal allergy testing may not be necessary for guiding implant selection.
Unicondylar knee arthroplasty using cobalt-chromium implants in patients with self-reported cutaneous metal hypersensitivity	Walker et al. [43]	No serious symptoms: at the mean follow-up of 3 years, no local or systemic symptoms of hypersensitivity to metal were reported. Survival rate: the survival rate of the implants at 3 years was 98.8% for revision for any reason and 97.6% for reoperations. Clinical outcome: the mean Oxford Knee Score (OKS) was 42.5, indicating good to excellent outcomes, with 59.7% of patients achieving excellent outcomes (OKS >41). Patient satisfaction: a high level of satisfaction with the prosthesis was reported, with a mean score of 0.61 on a scale from 0 (excellent) to 10 (completely dissatisfied).
Hypersensitivity reactions to metal implants: laboratory options	Carossino et al. [44]	The study found that the LTT was more effective than the PT in confirming metal allergies. Group 1 patients who had hypersensitivity underwent TKA with nonallergic implants and reported good outcomes at the 12-month follow-up, with no complications. In contrast, some patients in group 2A who underwent revision arthroplasty with nickel-free implants experienced significant pain relief, while others had persistent symptoms. The study highlights the importance of combining PT and LTT to accurately diagnose metal hypersensitivity and suggests that cytokine production monitoring may help predict clinical outcomes.
Sensitivity to implant materials in patients with total knee arthroplasties	Granchi et al. [41]	Frequency of positive skin reactions to metals increased significantly after TKA, either stable or loosened: no implant 20%; stable TKA 48.1%; loose TKA 59.6% (*P* = .001). TKA failure was 4-fold more likely in patients who had symptoms of metal hypersensitivity before TKA
Females with Unexplained Joint Pain Following Total Joint Arthroplasty Exhibit a Higher Rate and Severity of Hypersensitivity to Implant Metals Compared with Males Implications of Sex-Based Bioreactivity Differences	Caicedo et al. [45]	Metal sensitivity: females showed a significantly higher rate and severity of metal sensitization compared to males. Median lymphocyte stimulation index (SI) for males was 2.8, while for females it was 3.5 (*P* < .05). 49% of females had an SI of ≥4 (reactive), compared to 38% of males. Implant-related pain levels were also higher in females (mean pain score of 6.8) compared to males (mean score of 6.1, *P* < .0001).
Total knee arthroplasty in patients with hypersensitivity to metals	Innocenti et al. [46]	Hypersensitivity findings: out of 24 patients, 4 (16.6%) were identified as hypersensitive to metals. The overall incidence of actual sensitivity was 0.49% in the larger TKA population. Follow-up outcomes: the mean follow-up was 79.2 months. No patients reported hypersensitivity reactions or complications post-TKA. Pain levels (measured by the visual analog scale) improved significantly from a mean preoperative score of 7.2 to 1.8 postoperatively. The Knee Society Score and functional scores also showed significant improvement.
A Comparison of Clinical Outcomes After Total Knee Arthroplasty in Patients Who Have and Do Not Have Self-Reported Nickel Allergy: Matched and Unmatched Cohort Comparisons	Siljander et al. [47]	Clinical outcomes: there were no significant differences in clinical outcome scores (preoperative, 6 weeks, and 1 year) between the 2 groups. Revision rates: the revision rate was not significantly different between the 2 cohorts (3% for CoCr and 1% for nickel-free implants, *P* = .451). Survivorship free from revision was 94% for CoCr and 98% for nickel-free implants (*P* = .9). Postoperative reactions: the nickel-free implant cohort had a higher incidence of postoperative dermatitis, but no other significant differences in hypersensitivity manifestations were noted.
Do Patients With Hypoallergenic Total Knee Arthroplasty Implants for Metal Allergy Do Worse? An Analysis of Health Care Utilizations and Patient-Reported Outcome Measures	Tidd et al. [48]	Health care utilization: Average length of stay was similar for both groups (1.6 days each, *P* = .98). No significant differences in 90-day readmission rates (*P* = .89) or discharge dispositions (*P* = .82). Patient satisfaction: Satisfaction rates at 1 year were comparable: 84.1% for standard TKA and 80% for hypoallergenic TKA (*P* = .23). No significant differences in the achievement of Patient Acceptable Symptom State (PASS) or Minimal Clinically Important Difference (MCID) thresholds for KOOS pain and KOOS PS.
The association between metal allergy total knee arthroplasty, and revision	Münch et al. [49]	Prevalence of metal allergy: the study indicated that while overall metal allergy was not associated with a higher rate of revision, cobalt and chromium allergies were more frequently observed in patients requiring multiple revisions.
Metallic ion release after knee prosthesis implantation: a prospective study	Lons et al. [4]	This prospective study demonstrates a significant increase in blood levels of chromium, cobalt, and titanium 1 year after knee arthroplasty. Despite this increase, no correlation between metallic ion release and clinical scores or prosthetic volume was noted. The study highlights the importance of evaluating metallic ion levels as a potential diagnostic tool for complications following knee arthroplasty.
Metal hypersensitivity and metal ion levels in patients with coated or uncoated total knee arthroplasty: a randomized controlled study	Lutzner et al. [50]	The study found that total knee arthroplasties with both coated and uncoated implants did not lead to significant increases in metal ion levels or hypersensitivity reactions postoperatively. Although the findings are promising for the coated implant's safety, further long-term studies are needed to confirm these results and assess any potential benefits over time.
Patient-Reported Metal Allergy: A Risk Factor for Poor Outcomes After Total Joint Arthroplasty?	Nam et al. [19]	Patients reporting a metal allergy experienced significantly lower scores in various Knee Society Score subdomains (Function, Symptoms, Satisfaction, Expectation) compared to those without a reported metal allergy, indicating that metal allergy may be associated with poorer functional outcomes after total knee arthroplasty.
Prevalence of metal hypersensitivity in total knee replacement	Desai et al. [51]	Prevalence of metal hypersensitivity: 15.87% Most common allergen: chromium (11.58%) Significant symptoms associated with hypersensitivity: loss of function and patient dissatisfaction.
Screening for symptomatic metal sensitivity: a prospective study of 92 patients undergoing total knee arthroplasty	Niki et al. [52]	Prevalence of metal sensitivity: 26% of patients tested positive for sensitivity to at least one metal. Eczema incidence: 5.4% of patients developed eczema attributed to metal sensitivity post-TKA. Significant metal: chromium sensitivity was significantly associated with the development of eczema.
A prospective study concerning the relationship between metal allergy and post-operative pain following total hip and knee arthroplasty	Zeng et al. [53]	In TKA patients, the study concluded that metal allergy does not significantly affect postoperative pain levels, as evidenced by comparable VAS scores between metal allergy and nonmetal allergy groups. The results suggest that while metal allergies are prevalent, they may not directly contribute to the pain experienced postsurgery, and larger studies are needed to further clarify this relationship.
Nickel allergy does not correlate with function after total knee arthroplasty	Chimento et al. [15]	Nickel allergy prevalence: Among the 50 patients, 74% showed reactivity to nickel, making it the most common metal allergy identified. Reactivity scores: Nickel demonstrated higher reactivity compared to cobalt and chromium (*P* < .001). Females had a 3.41 times greater likelihood of exhibiting higher nickel reactivity compared to males (*P* = .0295). This study is the first to assess the correlation between nickel allergy and clinical outcomes in well-functioning TKA patients. The findings indicate that nickel allergy does not correlate with knee function postsurgery. Surgeons are advised to be cautious in attributing poor outcomes exclusively to metal allergies without further investigation into other potential causes.
Metal sensitivity in patients with orthopaedic implants: a prospective study	Frigerio et al. [54]	The findings suggest that relying solely on preoperative history-taking is insufficient for identifying metal sensitivity in TKA patients. The increase in metal sensitivity observed 1 year postimplantation implies that sensitization may occur due to the prosthesis itself. Initially, 22% (16 out of 72 patients who completed the study) tested positive for metal sensitivity before surgery. After 1 year postimplantation, the percentage of patients who tested positive increased to 29% (21 out of 72 patients).
Self-reported metal hypersensitivity in patients undergoing unicondylar knee arthroplasty":	Atilla et al. [59]	The findings indicate that self-reported metal hypersensitivity does not significantly affect functional outcomes or quality of life in patients undergoing UKA. This aligns with literature suggesting that metal hypersensitivity may not correlate with poor outcomes in knee arthroplasties. The study emphasizes the need for better identification of patients at risk of allergic reactions to metal implants, as current screening methods may not be sufficient.
No difference in patient reported outcome and inflammatory response after coated and uncoated total knee arthroplasty a randomized controlled study	Tille et al. [55]	The study found no significant increase in inflammatory response or differences in PROMs between coated and standard TKAs over a 3-year follow-up period. The inflammatory response appears limited, even in patients with known allergies. The coated implants did not result in higher complication rates or worse outcomes, suggesting they are a safe treatment option for patients who may require hypoallergenic implants.
Improved outcomes in patients with positive metal sensitivity following revision total knee arthroplasty	Zondervan et al. [56]	Patients with positive metal LTT sensitivity demonstrated significant improvements in pain and function following revision TKA to a hypoallergenic component. This study provides a treatment algorithm for managing patients with painful knee replacements, emphasizing the need for timely diagnosis and appropriate management strategies. They suggest that revision to hypoallergenic components can lead to improved outcomes.
Patch Test Results and Outcome in Patients with Complications from Total Knee Arthroplasty: A Consecutive Case Series	Sasseville et al. [57]	Hypersensitivity to implant components may contribute to complications in TKA, but the study did not establish a definitive correlation between hypersensitivity and surgical outcomes. Preoperative testing is recommended for patients with known allergies to potentially avoid postoperative complications.
Lymphocyte Transformation Testing (LTT) in Cases of Pain Following Total Knee Arthroplasty Little Relationship to Histopathologic Findings and Revision Outcomes	Yang et al. [30]	63% cases showed fibrosis, lymphocytic infiltration with ALVAL score 3.1 ± 1.9 (maximum 10). No correlation between ALVAL score and LTT testing. Improved outcome scores postrevision were seen
What role does metal allergy sensitization play in total knee arthroplasty revision?	Lionberger et al. [58]	Ratio of CD4+/CD8+ T-lymphocytes was 1.28 in nickel-sensitive patients vs 0.76 in the control (*P* = .009). The study provides histological evidence supporting the presence of nickel allergy sensitization in patients with failed arthroplasties, even when clinical and radiographic abnormalities are not evident. The findings suggest that evaluating CD4+/CD8+ cell counts may help identify nickel sensitization and encourage the use of low-nickel implant designs for these patients. No difference in functional or clinical outcomes after revision

KOOS PS, Knee Injury and Osteoarthritis Outcome Scores - Physical Function Shortform.

There were an array of clinical tests used to assess for the presence of MH. Nickel, cobalt, and chromium were the most commonly assessed metals among all the studies, as they reflect the literature in being the most common metals to be sensitized to.

SPT and LTT were the most frequent diagnostic methods used, either alone [15,27,41,45,49,53,56] or in conjunction with other testing for added sensitivity/specificity. Innocenti et al. [46] along with the use of SPT and LTT used enzyme-linked immunosorbent assay and confocal microscopy, respectively, to analyze cytokine expression to help evaluate the immune response to the metal implants and to examine if any abnormalities in CD3 and CD4 following contact with metal ions. Lons et al. [4] looked at metal-ion level on serology pre- and post-TKA implant noting that there was a significant increase in blood levels of chromium, cobalt, and titanium 1 year after knee arthroplasty but despite this increase, no correlation between metallic ion release and clinical scores or prosthetic volume was noted. Lützner et al. [50] in a similar way looked at metal ion levels but used this in conjunction with SPT. Their results differed from Lons et al. [4] as they saw no significant increase in metal ions 12 months postimplantation of either a coated or uncoated TKA. Interestingly, Tille et al. [55] recently hypothesized that MH would be associated with a local inflammatory reaction around a neo-synovium and included serological samples to assess cytokine levels. The study found no significant increase in inflammatory response or differences in PROMs between coated and standard TKAs over a 3-year follow-up period with the inflammatory response appears limited, even in patients with known allergies. Finally, 2 studies [30,58] incorporated histological samples into their analysis which were obtained during revision TKA (rTKA) procedures. Lionberger et al. [58] noted on synovial biopsies, an increased ratio of CD4+/CD8+ T lymphocytes being increased to 1.28 in nickel-sensitive patients vs 0.76 in the control (*P* = .009) with the authors commenting that this provides some histological evidence supporting the presence of nickel allergy sensitization in patients with failed arthroplasties. This, however, is contrasted by Yang et al. [30] who showed that despite an elevated LTT stimulation index of a mean 9.4 ± 8.2, there was a low corresponding Aseptic Lymphocyte-Dominant Vasculitis Associated Lesions (ALVAL) score indicating a low immune reaction.

This, very importantly, highlights that a positive SPT or LTT does not necessarily indicate that an immune response is the cause of pain and stiffness after TKA.

### Patient-reported outcome scores

A multitude of PROMs were utilized throughout this literature set. KSSs were used in 6 studies [19,27,42,45,46,50], Oxford Knee Scores (OKSs) in 3 studies [15,43,55], Knee Injury and Osteoarthritis Outcome Scores (KOOS) in 2 studies [47,48], A Knee Society Clinical Rating system in 2 [41,58], and a visual analog scale in 4 studies [47,53,56].

The outcomes for the use of KSS scoring differed across the 6 publications. Schmidt et al. [42], looking at early outcomes post-TKA, found no significant differences in KSS-Function (KSS-F) and KSS-Knee (KSS-K) between patients with SRMA and those without. Stiffness, physical function, and knee flection were comparable across both groups with regard to early postoperative outcomes. Bravo et al. [27] looked at the risk of arthroplasty failure in patients with positive SPT and had no statistically significant differences in postoperative pain scores with 86% of SPT-positive patients reporting none to mild pain, while 14% had moderate to severe pain. In comparison, 91% of SPT− patients reported none to mild pain, and 9% had moderate to severe pain. KSS-F scores from preoperative to postoperative were similar across all groups, indicating no significant difference in functional improvement based on skin patch test results.

Lutzner et al. [50], in their randomized controlled trial investigating MH and metal ion levels in patients with coated or uncoated TKA, highlighted no significant difference between the groups, with both showing improvements in pain scores, KSS-F and KSS-K at 3 months and 1 year.

Despite MH being significantly more prevalent in the female gender, Caicedo et al. [45] from their population of MH patients (n = 496) found the KSS-F and KSS-K scores did not significantly differ between males and females.

Only Nam et al. [19] demonstrated a statistically significant poorer association with MH and KSS scores compared to non-MH in patients who have undergone TKA.

OKSs were utilized in 3 studies [15,43,50,55]. Walker et al. [43] specifically looked at cobalt chromium unicondylar knee implants in patients with a self-reported MH and found that the overall mean OKS: 42.5 (standard deviation: 2.5; range: 37 to 48) with no statistically significant difference in OKS based on type of knee arthroplasty, gender, or hypersensitivity status (*P* > .05). Chimento et al. [15] report no significant correlation found between metal reactivity and OKS scores for nickel (rho = −0.1779), cobalt (rho = −0.0036), or chromium (rho = −0.1748) across their study population, as well as when stratified by gender. Tille et al. compared coated and standard TKA regarding inflammatory response and patient-reported outcomes. In this study, after a 3-year follow-up period, OKS scores in the metal allergy group (36.1 ± 7) were comparable to those in the standard and coated TKA groups (38.5 ± 8.1 and 37.9 ± 8, respectively) and this was echoed in Lützner et al.’s randomized controlled trial in which there was a significant improvement in the OKS scores from preoperative to the 1-year follow-up for both groups (coated vs uncoated TKA) indicating enhanced knee function and quality of life post-TKA.

KOOS were overall positive in the 2 studies that used this PROM. Siljander et al. retrospectively reviewed 282 patients with a nickel allergy identifying those who received a cobalt chrome (CoCr) implant and those who had a nickel-free implant. There were no significant differences in KOOS scores between the CoCr implant and nickel-free implant groups at any time point (preoperative, 6 weeks, and 1 year). Preoperative Knee Injury and Osteoarthritis Outcomes Scores (KOOS JR: CoCr implant: 50.2 and with nickel-free implant: 50.6 and at 1 year the KOOS JR: CoCr implant scored and mean 72.8 while nickel-free implants scored an average of 74. Both cohorts showed significant improvements in KOOS scores from preoperative to 1-year postsurgery, indicating effective outcomes for both implant types despite the presence of nickel allergy. Tidd et al. also demonstrated there were no significant differences in KOOS pain scores in their MH cohort compared to non-MH cohort after 1 year between those receiving hypoallergenic and standard TKA (KOOS standard 82.1/KOOS hypoallergenic 81.9)

Visual analogue scale (VAS) were a frequent outcome measure seen in this systematic review with 4 studies including them as PROMs [47,53,56].

VAS scores improved across all studies following primary or revision knee arthroplasty regardless of MH status. Siljander et al. in their randomized controlled trial comparing primary knee arthroplasty with nickel-free implants or standard CoCr, highlighted a comparable sequential improvement in VAS scores across both groups from preoperative to 1 year postoperative. Similarly, Zeng et al. found no statistically significant difference in VAS scores between the metal allergy group and the non-metal allergy group for both THA and TKA patients (*P* > .05). Zondervan et al. showed that MH patients (‘reactive patients’) being revised to a “hypoallergenic” implant experience a significant reduction in pain levels, while nonreactive patients show only moderate improvement, suggesting a relationship between metal sensitivity and postoperative pain. This difference, however, was not statistically significant. Finally, Atilla et al. demonstrated that at the terminal follow-up, the mean EQ-5D-3L VAS score was 87.67 ± 9.58 in the no-MH group and 85.15 ± 8.68 in the MH group with no statistical significance (*P* > .05).

### Revision and failure in primary TKA

There were 9 studies reporting revision rates in patients undergoing primary TKA [27,42,43,[46], [47], [48], [49],^52^,^53^] with either a self-reported MH (SRMH) or confirmed hypersensitivity on testing. The pooled population of which is 1165 MH patients with a mean follow-up of 36 months (range, 12-79). The total all*-cause* number of revisions documented was 44 giving an *all-cause* revision rate of 3.7% (ie, due to infection, periprosthetic fracture, aseptic loosening, etc.). Most studies concluded that there was minimal evidence to suggest there be a causal relationship between MH and rTKA procedures. There was a scarcity of reporting on specifically “aseptic loosening,” which is classically associated with a potential MH reaction. Only Granchi et al. [41] commented on 21 cases of aseptic loosening with positive SPT with the proportion of SPT positive results being higher in patients with aseptic loosening than other cohorts. They, however, did not report revision procedures in their paper.

### Outcomes of metal hypersensitivity and revision TKA

Four studies included specifically looked at rTKA [30,[56], [57], [58]] with a total population of 147 knees, an average age of 63 years, a 60% female dominance, and a mean follow-up time of 23.75 months.

Zondervan et al. in their single-center retrospective cohort study included 46 patients who underwent revision surgery and who had been assessed for MH. MH testing was only availed of following exclusion of all other causes for a poorly functioning/unsatisfied patient post primary TKA. Out of the total 46 patients included, 39 patients were found to be MH on LTT. All these patients were revised with a “hypoallergenic” implant. Outcome measures included a documented preoperative and postoperative range of motion, a Likert scale for satisfaction, and a Pain Intensity Numerical Rating Scale. Patients with positive metal LTT sensitivity demonstrated significant improvements in pain and function following rTKA to a hypoallergenic component with the authors suggesting that revision to hypoallergenic components in this cohort can lead to improved outcomes. Of note, there were over 15% patients lost to follow-up, and a statistical analysis was unable to be performed.

Sasseville et al. discussed that hypersensitivity to implant components may contribute to complications in TKA, but the study did not establish a definitive correlation between hypersensitivity and surgical outcomes in their study.

Yang et al. interestingly sampled neo-synovial tissue during the revision procedures of their 27 patients who had an associated positive LTT. Despite histopathology showing higher levels of fibrosis and lymphocytic infiltration in only 63% of cases, clinically KSS scores improved postoperatively leading the authors to comment that no conclusive correlation was found between LTT results, ALVAL scores, or clinical outcomes.

Finally, Lionberger et al. included 32 symptomatic pTKA following exclusion for other modes of failure. LTT testing was conducted with 19 patients being positive and 13 being negative. They also gauged histopathological responses in neo-synovial tissue taken at the time of revision surgery to a ceramic coated “hypoallergenic” implant. Interestingly, there was a clear difference in the ratio of CD4+/CD8+ T-lymphocytes in nickel-sensitive patients (1.28) vs 0.76 in the control (ie, LTT negative) (*P* = .009). This study provides histological evidence supporting the presence of nickel allergy sensitization in patients with failed/symptomatic arthroplasties, even when clinical and radiographic abnormalities are not evident. The findings suggest that evaluating CD4+/CD8+ cell counts may help identify nickel sensitization and encourage the use of low-nickel implant designs for these patients. However, again, no difference in functional or clinical outcomes after revision was seen.

### Outcomes in unicondylar knee replacement

Two studies included looked at outcomes following UKA [43,59] with a total population of UKA of 218 knees, an average age of 62.35 years, and a 91.6% female predominance. Both studies use SRMA as the mode for diagnostic inclusion. A total of 95 patients had a SRMA prior to UKA between the 2 studies. Both studies utilized the same Oxford (Zimmer Biomet) brand of UKA implant.

Walker et al. included a total of 82 patients out of 1737 suitable for medial UKA reporting cutaneous MH to cobalt, chromium, or nickel. At the mean follow-up of 3 years, no local or systemic symptoms of hypersensitivity to metal were reported.

The survival rate of the implants at 3 years was 98.8% for revision for any reason and 97.6% for reoperations with a mean OKS of 42.5, indicating good to excellent outcomes, with 59.7% of patients achieving excellent outcomes (OKS >41). Finally, they reported a high level of satisfaction with the prosthesis with a mean score of 0.61 on a scale from 0 (excellent) to 10 (completely dissatisfied).

Atilla et al. included 136 patients with 10% (13 patients) self-reporting MH prior to surgery. Their findings align with Walker et al. in that self-reported MH does not significantly affect functional outcomes or quality of life in patients undergoing UKA. Both cohorts in their study showed equivalent Western Ontario and MacMaster Universities Osteoarthritis Index (WOMAC) and VAS scores with no statistically significant change in serological analysis of eosinophil count at 18 months.

## Discussion

This study provides a comprehensive review of the literature regarding outcomes in knee arthroplasty patients with MH. Included is 24 high-quality methodological studies, in which there are 5615 pooled knee arthroplasty cases and 1849 patients with an MH diagnosis (based on individual study diagnostic criteria). As such, despite significant heterogeneity in this literature, trends in study outcomes and discussions can be logically interpreted.

Looking at demographics, a clear sexual dimorphism favoring females among all allergens is well described [18,22,45,60]. Atopy and patient-reported allergies are a known risk for worse satisfaction and WOMAC functional scores post-TKA [12,13] and that is seen with regard MH with this review. Schmidt et al [42], Caicedo et al [45], and Nam et al. [19] allude to increased pain scores (WOMAC) in females with SRMH or LTT positivity, respectively.

However, attributing all these poor outcomes of knee arthroplasty to an MH alone proves difficult, as is clearly demonstrated by the fact that clinically the majority of patients with SRMH, SPT-positive testing, LTT-positive testing, or both have statistically significant improved pain scores and PROMs [15,27,43,46,47,53].

Metal sensitization may occur, but there is certainly a lack of data to truly support its overall impact. Lons et al. highlighted the significant increase in cobalt, chromium, and titanium levels in the blood after 1 year of TKA implantation and concluded that this could contribute to MH reactions. Frigerio et al. did demonstrate an increase in the number of positive SPT 1 year post implantation of TKA (n = 5), and Lionberger et al. in a revision setting, concluded that the increase in the neo-synovial ratio of CD4+/CD8+ T-lymphocyte supports the evidence of nickel hypersensitivity reactions. But, Yang et al. despite showing fibrosis and lymphocytic infiltrates on neo-synovial biopsy, showed that no correlation could be drawn between clinical symptoms, LTT, and ALVAL biopsy scores.

With conflicting literature, there has to be a pragmatic approach to the “at-risk” patient for MH.

In terms of who to test/when to test for MH, there has been two consensus statement looking at this specific question. Razak et al. [61] in a UK-based Delphi Study, showed that 17 surgeons had consensus (>60% agreement) in that even if a metal allergy is suspected, a patch test is not necessary to be done to confirm the presence of a metal allergy. This is mirrored by a German-centered, multinational, multispecialty Delphi study of 12 experts in which mild symptoms of a SRMH did not reach consensus for testing preoperatively for TKA; however, severe symptoms did reach consensus for preoperative MH testing [62]. Neither study showed a need for routine preoperative testing in patients undergoing knee arthroplasty.

With regard to implant choice, Xie et al. [63] conducted a systematic review of these “hypoallergenic implants” and concluded that no significant differences lie between hypoallergenic and standard TKAs with overall good survival rates. In the previously alluded to Delphi study, Razak et al. had consensus on using standard CoCr implants in patients patch testing positive for cobalt, chromium, or nickel. This conclusion stems from the systematic review and meta-analysis conducted by Granchi et al. [14], in which the positive predictive value of MH testing in patients undergoing joint arthroplasty was not statistically proven, even though the paper suggested that patients with a history of metal allergy should be patch tested.

There are an array of “hypoallergenic implants” on the market. Commonly available products are summarized in Table 4. Despite an added cost, in a patient with a strong history of MH, there appears to be no compromise in terms of prosthesis performance, outcomes, and longevity for the patient.

**Table 4:** TKA and hypoallergenic implants available.

Manufacturer	Implant name	Tibial component	Femoral component	ODEP rating
DePuy	*PFC Sigma* *LCS Complete* *Attune*	Complete zirconia nitride Complete zirconia nitride N/A	Complete zirconia nitride Complete zirconia nitride N/A	15A 15A 5A∗
Smith & Nephew	*Journey II* *Genesis II*	Oxidized zirconium (Oxinium) titanium	Oxidized zirconium (Oxinium) Oxidized zirconium	7A∗ 15A
Stryker	*Triathlon*	Titanium nitride	Titanium nitride	15A
Zimmer Biomet	*Vanguard* *Nexgen* *Persona* *Oxford UKA TiNbN*	Titanium niobium nitride coating Titanium niobium nitride coating N/A Titanium niobium nitride	Titanium niobium nitride coating Titanium niobium nitride coating N/A Titanium niobium nitride	13A 15A 5A∗ 10B
B Braun & Aescap	*Columbus AS*	Zirconium nitride coating of standard implant	Zirconium nitride coating of standard implant	10A

ODEP, Orthopaedic Data Evaluation Panel Score.

The asterisk (∗) represents the 'star' in ODEP rating.It is an additional part of the rating system which denotes a benchmark replacement rate of less than 5% at 10 years.

While using this “hypoallergenic prosthesis,” it is important to note that standard “used” instrumentation has been shown to contribute to a significant amount of free metal debris in the joint. Gotterson et al. [64] performed bony resections simulating TKA in a pig model and collected metal debris from sawblades, cutting blocks, and bone surfaces, showing an average of 1.13 mg of metal lost from the sawblades alone, with cutting blocks contributing to even more metal debris. It is estimated that millions of metal particles are generated during standard TKA resections. This was subsequently tested in vivo by Lawrie et al. [65] looking at knee joint fluid pre- and post-TKA showing that there were substantial levels of nickel generation while performing a “nickel-free” TKA.

**Figure 2:**
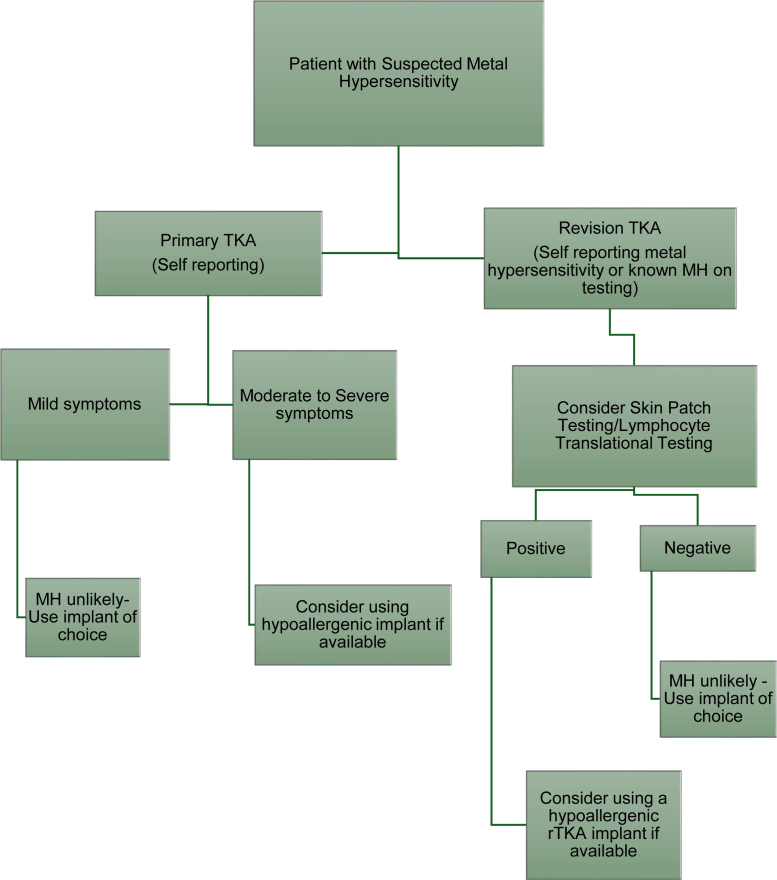
Clinical algorithm for metal hypersensitivity.

In the rTKA setting, if all other causes for pain, implant loosening, bony lysis, and failure have been excluded, then MH testing seems logical. Based on this systematic review, it is of the authors’ opinion that both SPT and LTT should be conducted despite knowing the limitations of the testing. One could consider a synovial biopsy prerevision, but this is an invasive procedure that carries its own risk of prosthetic joint infection in an otherwise aseptic, unhappy knee. If a patient has SPT and LTT positive results with/without a history of metal allergy, one could consider the use of a “hypoallergenic implant” (See Figure 2).

## Conclusions

This systematic review highlights the discrepancy between patient symptomatology, diagnostic testing, and clinical outcomes in suspected cases of MH. Outcomes are likely multifactorial and having an SRMA or positive skin patch test/lymphocyte transformation test does not appear to have a clinically significant impact on pain and functional scores post knee arthroplasty. More robust registry data are needed to truly understand the extent of the problem, as it is likely underreported, and comparable clinical research is needed to understand better how to manage MH in knee arthroplasty.

## Conflicts of interest

E. Servien receives royalties from Smith & Nephew and is on the speakers bureau/paid presentations for Smith & Nephew; all other authors declare no potential conflicts of interest.

For full disclosure statements refer to https://doi.org/10.1016/j.artd.2026.102003.

## CRediT authorship contribution statement

**Ryan Roopnarinesingh:** Writing – original draft, Methodology, Formal analysis, Data curation. **Elvire Servien:** Writing – review & editing, Supervision, Conceptualization. **James Harty:** Writing – review & editing, Supervision, Conceptualization.

## Appendix A Supplementary data

Conflict of Interest Statement for Servien

Conflict of Interest Statement for Harty

Conflict of Interest Statement for Roopnarinesingh
